# From Gene Function to Precision Intervention: CRISPR/Cas9 and Stem Cell-Based Strategies as Emerging Disease-Modifying Approaches in PMOS

**DOI:** 10.1007/s12015-026-11188-4

**Published:** 2026-07-07

**Authors:** Masuma Khatun, Karolina Lundin, Timo Tuuri, Terhi Piltonen, Juha S. Tapanainen, Andres Salumets

**Affiliations:** 1 Department of Obstetrics and Gynecology, University of Helsinki and Helsinki University Hospital, Haartmaninkatu 2, 00290 Helsinki, Finland; 2 Department of Obstetrics and Gynecology, Institute of Clinical Medicine, University of Tartu, Tartu, Estonia; 3 Department of Obstetrics and Gynecology, Clinical Research Unit, Medical Research Center Oulu, University of Oulu and Oulu University Hospital, Oulu, Finland; 4 Celvia CC, Tartu, Estonia; 5 Department of Clinical Science, Intervention and Technology, Karolinska Institute and Karolinska University Hospital, Stockholm, Sweden

**Keywords:** Polyendocrine metabolic ovarian syndrome, Stem cells, CRISPR/Cas9, Gene editing, Induced pluripotent stem cells (iPSCs), Mesenchymal stem cells, Disease modelling, Translational medicine

## Abstract

Polyendocrine metabolic ovarian syndrome (PMOS) is a complex endocrine–metabolic disorder affecting up to 18% of women worldwide and remains the leading cause of anovulatory infertility. Despite extensive research, current treatments primarily target symptoms, including menstrual irregularities, hyperandrogenism, and metabolic dysfunction, without addressing the underlying molecular and tissue-level disturbances. Advances in multi‑omic profiling have identified disruptions across neuroendocrine, metabolic, inflammatory, and extracellular matrix pathways, alongside genetic susceptibility at loci such as *DENND1A*, *CYP17A1*, *LHCGR*, *FSHR*, *IRS1*, and *PPARG*. However, the functional roles of many variants remain unresolved. CRISPR/Cas9 gene editing enables precise interrogation of these pathways, while stem cell–based platforms, including mesenchymal stem cells (MSCs), exosomes, and gene-edited induced pluripotent stem cells (iPSCs), may serve as complementary platforms for regeneration and disease modeling. Preclinical studies demonstrate that MSCs and their derivatives modulate inflammation, restore ovarian structure, and improve metabolic parameters, while iPSC-based models enable patient-specific investigation of steroidogenic and metabolic abnormalities. Translational challenges remain, including targeted delivery, off-target effects, phenotypic heterogeneity, and regulatory considerations. Integrating CRISPR‑based functional genomics with stem cell research may shift PMOS management from symptom‑focused care to targeted, mechanism‑driven interventions that could modify the course of PMOS (Graphical Abstract).

**Graphical Abstract:**

Mapping the paradigm shift from symptomatic management to mechanism-driven precision medicine in Polyendocrine Metabolic Ovarian Syndrome (PMOS). The left panel summarizes current clinical challenges defined by purely symptomatic treatments for anovulatory infertility, hyperandrogenism, and metabolic dysfunction, a limitation sustained by a historically poor understanding of underlying disease mechanisms. The middle panel outlines novel biotechnological platforms for mechanistic discovery and therapeutic intervention. Gene function interrogation uses CRISPR/Cas9 editing to target specific risk loci, including *DENND1A*, *CYP17A1*, *IRS1*, and *PPARG*, to dissect functional pathways governing androgen excess, follicular arrest, and insulin resistance. Stem cell-based strategies employ mesenchymal stem cells (MSCs) and MSC-derived exosomes to modulate systemic inflammation, restore ovarian physiological function, and improve homeostatic metabolic parameters. Patient-specific disease modeling utilizes induced pluripotent stem cells (iPSCs) differentiated into granulosa-like cells or in vitro endometrial models to recapitulate distinct steroidogenic and metabolic abnormalities. The right panel illustrates the ultimate clinical goal of these integrated approaches, demonstrating how transitioning to targeted therapies establishes a balanced endocrine-metabolic profile, improves reproductive function, and reduces long-term metabolic risks, successfully moving the field toward mechanism-driven care and precision medicine. This figure was created with BioRender.com.

**Figure:**
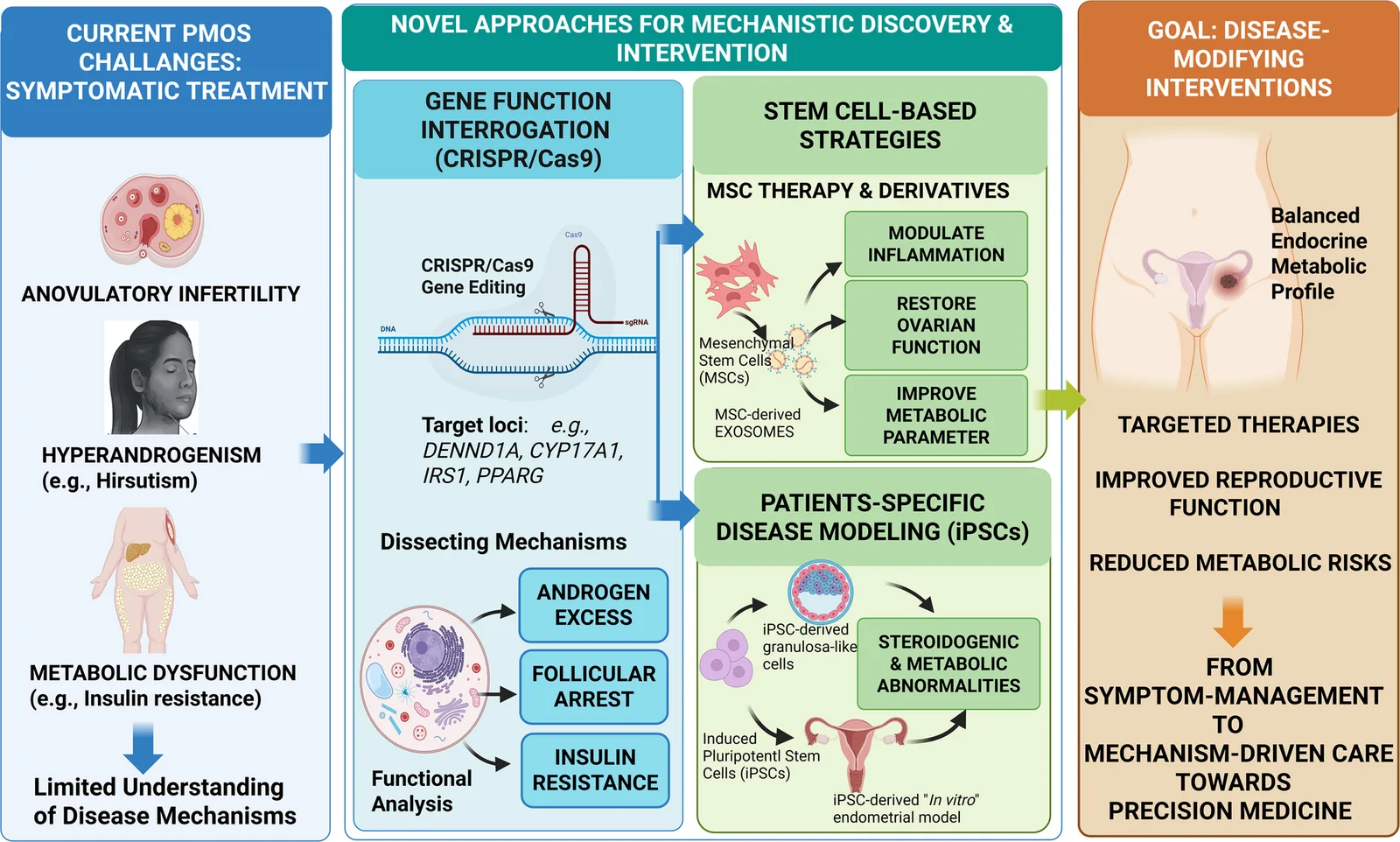

## Introduction

Polyendocrine metabolic ovarian syndrome (PMOS), previously known as polycystic ovarian syndrome (PCOS), is a multifaceted endocrine–metabolic disorder affecting ~ 5–18% of women worldwide, depending on ethnicity and diagnostic criteria, and accounts for up to 70–80% of anovulatory infertility cases [1–3]. PMOS likely stems from ancient genetic and epigenetic adaptations that once conferred evolutionary advantages but now predispose women to a broad spectrum of reproductive, metabolic, and psychological complications [4–6]. According to the 2023 international guidelines, PMOS diagnosis requires at least 2 of the following criteria: hyperandrogenism (HA, clinical or biochemical), irregular cycles, or polycystic ovarian morphology (PCO ≥ 20 follicles and/or ovarian volume ≥ 10 mL), excluding related conditions [7].

PMOS is frequently accompanied by overweight and/or obesity (OB; 38–88%) [8], insulin resistant (IR; 35–80%) [9], HA-induced immune alteration [10], increased risk of cardiovascular disease (CVD; 48–150%) [11], dyslipidemia (40–70%) [12], depression and anxiety (28–76%) [13], metabolic- dysfunction-associated steatotic liver disease (MASLD; 34–70%) [14], endometrial cancer [15], and type 2 diabetes mellitus (T2DM) [16].

Despite extensive research, current therapies remain largely symptomatic, focusing on menstrual regulation and androgen suppression rather than targeting the underlying neuroendocrine, metabolic, or molecular disturbances driving disease progression [17, 18]. Clinical heterogeneity further complicates management, limiting personalized treatment and leaving central pathogenic mechanisms, such as disrupted folliculogenesis, hypothalamic-pituitary dysregulation, ovarian dysfunction, and genetic or epigenetic determinants, poorly understood [19]. Unsupervised clustering analyses of clinical variables across five international cohorts have identified four reproducible PMOS subtypes: HA-PMOS, OB-PMOS, Sex hormone-binding globulin (SHBG)-PMOS, and Luteinizing hormone (LH)-PMOS. Each subtype exhibits distinct reproductive and metabolic profiles and differential responses to in vitro fertilization, supporting a biologically informed stratification framework [20].

These limitations have stimulated interest in next-generation therapeutic approaches. Stem cell–based strategies are being explored for their potential to promote ovarian tissue repair and partial endocrine restoration, while CRISPR/Cas9 technologies are primarily used as research platforms to interrogate PMOS-associated genetic variants. Beyond therapeutic prospects, both approaches may facilitate disease modeling, drug discovery, and precision medicine development [21, 22].

This review integrates current understanding of PMOS pathophysiology with the evolving applications of stem cell-based therapy and CRISPR/Cas9 gene editing, highlighting their potential to advance personalized, disease-modifying interventions.

## Multi-Omic Landscape of PMOS Pathophysiology

**Fig. 1:**
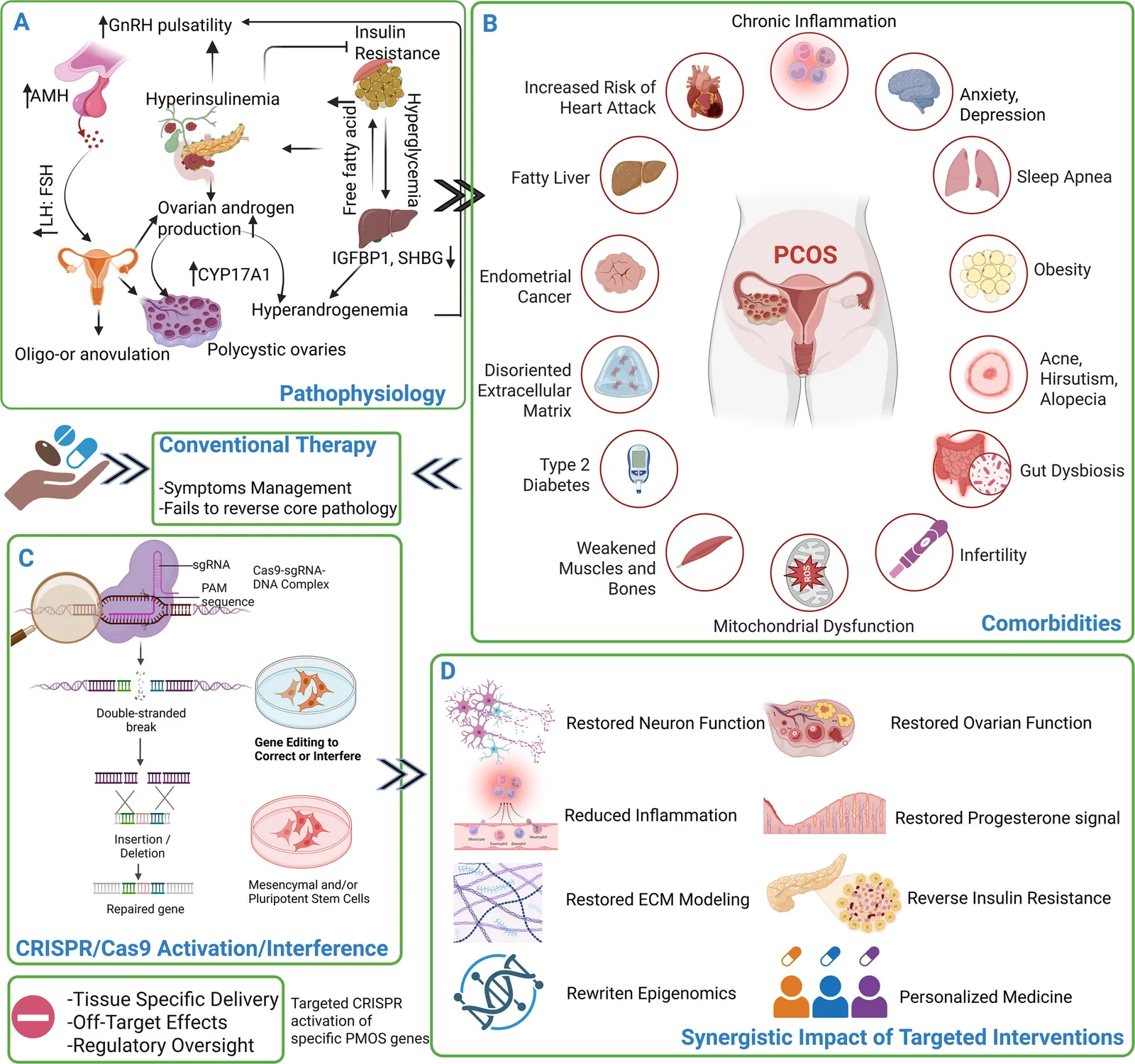
The complex pathophysiological landscape of Polyendocrine Metabolic Ovarian Syndrome (PMOS) and the shift toward CRISPR/Cas9 and stem cell-mediated targeted interventions. **A** Pathophysiology. Schematic representation of the neuroendocrine and metabolic feedback loops driving PCOS. Elevated gonadotropin-releasing hormone (GnRH) pulsatility and increased anti-Müllerian hormone (AMH) levels disrupt the LH:FSH ratio, promoting ovarian androgen production via upregulated enzyme activity (e.g., CYP17A1). Concurrently, hyperinsulinemia, driven by insulin resistance, exacerbates hyperandrogenemia directly and indirectly by decreasing hepatic production of IGFBP1 and SHBG, leading to elevated free fatty acids, hyperglycemia, oligo- or anovulation, and a polycystic ovarian morphology. **B** Comorbidities. Systemic clinical manifestations secondary to chronic inflammation and metabolic/hormonal dysregulation in PCOS, including increased risk of myocardial infarction, fatty liver disease, endometrial cancer, disorganized extracellular matrix (ECM), type 2 diabetes, musculoskeletal weakening, mitochondrial dysfunction, infertility, gut dysbiosis, dermatological symptoms (acne, hirsutism, alopecia), obesity, sleep apnea, and psychological disorders (anxiety, depression). Conventional therapies (top left) broadly manage these symptoms but fail to reverse the underlying core pathology. **C** CRISPR/Cas9 Activation/Interference. A precision medicine framework utilizing guide RNA (sgRNA) and Cas9 complexes to induce targeted double-stranded breaks, insertions/deletions, or genomic activation/interference in specific disease-associated loci. This gene-editing platform can be combined with mesenchymal stem cells (MSCs) or pluripotent stem cells for functional correction. Box (bottom left) highlights current translational challenges, including tissue-specific delivery, off-target risks, and regulatory oversight. **D** Synergistic Impact of Targeted Interventions. Therapeutic outcomes of combining precision gene editing with stem cell platforms, demonstrating restored neuroendocrine and ovarian function, attenuated chronic inflammation, rescued progesterone signaling, normalized extracellular matrix (ECM) remodeling, reversal of insulin resistance, and rewritten epigenomics to achieve true personalized medicine. This figure was created with BioRender.com

PMOS arises from the intersection of neuroendocrine disruption, metabolic dysfunction, chronic inflammation, and genetic susceptibility. At the neuroendocrine level, increased gonadotropin-releasing hormone (GnRH) pulsatility elevates the LH/follicle-stimulating hormone (FSH) ratio, promoting ovarian androgen excess and impairing follicular maturation [23]. Elevated Anti-Müllerian hormone (AMH) levels, produced by the numerous small antral follicles, further amplify GnRH activity while reducing FSH sensitivity and aromatase activity [24]. Concurrently, IR and compensatory hyperinsulinemia exacerbate LH-driven androgen production and suppress SHBG, reinforcing a self-perpetuating endocrine-metabolic cycle [25]. The pathophysiology and associated comorbidities of PMOS are illustrated in Fig. 1A, B.

### Tissue-Level and System Biology Insights

Beyond ovarian function [26], multi-omic investigations reveal widespread systemic alterations [27]. Single-cell transcriptomics present altered uterine cellular composition and gene expression patterns that impair endometrial receptivity, particularly in hyperandrogenic and IR phenotypes [28–31].

Gut microbiome analyses consistently show reduced microbial diversity and enrichment of pro-inflammatory taxa (e.g., decreased *Bifidobacterium* and altered *Firmicutes/Bacteroidetes* ratio) contributing to enhanced intestinal permeability, systemic inflammation, and metabolic dysfunction [32, 33].

PMOS is also increasingly recognized as a disorder of dysregulated extracellular matrix (ECM) remodeling. Mechanisms include inflammation-induced Lysyl oxidase (LOX) overexpression, advanced glycation end product (AGE)-mediated collagen deposition, disrupted TGF-β signaling, and altered actomyosin contractility. These changes impair follicular development and oocyte competence [34]. Key pathways implicated include IL-1β–ERK1/2–JNK–cJun–LOX, AGE–pERK–NF-κB–LOX, TGF-β, NLRP3, PI3K/AKT, and Hippo/YAP, all of which regulate ECM structure, fibrosis, and oocyte maturation [35, 36].

Mitochondrial dysfunction represents another central feature, characterized by reduced mitochondrial DNA copy number, pathogenic variants, and epigenetic dysregulation. These abnormalities are closely linked to IR, chronic inflammation, adipocyte dysfunction, impaired folliculogenesis, oxidative stress, and cardiometabolic comorbidities [37]. Further bioinformatic integration has identified mitochondrial dysfunction-related hub genes such as *LIFR*, *PBK*, *PRKAA1*, *RCAN1*, and *MMP9* as potential regulators [38].

### Genetic Architecture and Causal Inference

PMOS is a highly heritable polygenic condition, with heritability estimates approaching 70% [39, 40] [41]. Genome-wide association studies (GWAS) and Mendelian Randomization (MR) analyses have identified ~ 100 susceptibility loci linked to gonadotropin regulation, ovulatory dysfunction, insulin signaling, and androgen biosynthesis [42–46]. MR studies further support causal roles for elevated BMI, IR, and testosterone levels in PMOS development [47, 48].

Although typically polygenic, rare monogenic or oligogenic forms have been reported. These include pathogenic variants affecting Insulin signaling, such as *INSR*, leading to severe IR [49, 50]. Moreover, mutations in steroidogenesis enzymes, including *CYP17A1*, *CYP11A1*, and *HSD3B2,* impair ovarian and adrenal steroid biosynthesis [51]. Variants in androgen synthesis regulators, such as *DENND1A,* further highlight a mechanistically coherent subgroup that may theoretically benefit from targeted gene-editing strategies [52]. Nevertheless, classical genetic variants explain only ~ 10% of PMOS heritability [53, 54], underscoring the importance of epigenetic regulation, gene–environment interactions, and mitochondrial epigenomics—particularly in obese, IR women seeking fertility preservation [55, 56]. Key susceptibility loci and their corresponding candidate genes with proposed mechanistic roles are summarized in Table 1.

**Table 1:** Key PMOS-associated GWAS-derived genetic loci, their primary molecular functions, biochemical pathways, and pathophysiologic relevance

Gene/Locus	Primary Molecular Function	Pathway(s)	Specific PMOS Relevance	Reference
*DENND1A* (9q33.3)	Regulates endosomal trafficking; enhances vesicular signaling	Androgen biosynthesis pathway	Increases theca-cell androgen production via DENND1A.V2 isoform, driving hyperandrogenism and anovulatory cycles	[52, 57]
*LHCGR* (2p16.3)	Affects LH/CG signaling; regulates ovarian steroidogenesis	Gonadotropin signaling	Alters LH responsiveness, disrupting normal ovulation, and promoting follicular arrest	[58, 59]
*FSHB* (11p14.1)	Encodes FSH β-subunit; required for FSH synthesis	Gonadotropin signaling	Alters circulating FSH levels, directly impairing follicular maturation and menstrual irregularities	[60, 61]
*THADA* (2p21)	Regulates cellular apoptosis and systemic energy homeostasis	Insulin signaling/Metabolic pathways	Associated with IR, obesity, and T2DM; exacerbates severe metabolic PMOS phenotype	[57, 62]
*HMGA2* (12q13.2)	Regulate chromatin remodeling, granulosa cell growth, and differentiation	Growth and developmental regulation	Linked to early menarche; drives increased ovarian volume and altered ovarian morphology	[63, 64]
*INSR*	Regulate insulin receptor signaling; glucose uptake	Insulin signaling	Reduces insulin sensitivity; compensatory hyperinsulinemia amplifies ovarian androgen production	[65, 66]
*YAP1*	Regulate Hippo pathway; cell proliferation and apoptosis	Cell growth regulation; Ovarian folliculogenesis	Disrupts granulosa cell function, follicular development, and metabolic traits	[67, 68]
*CYP11A1, CYP17A1, CYP19A1*	Catalyzes sequential steroidogenesis enzymatic steps	Androgen and estrogen biosynthesis	Upregulates autonomous androgen production in theca cells, contributing to PMOS hyperandrogenism	[51, 69]
*HSD17B5 (AKR1C3)*	Converts weaker androstenedione into active testosterone	Androgen biosynthesis	Enhances localized intraovarian and adrenal testosterone production rates	[70, 71]
*FTO*	RNA demethylase regulating energy intake and adiposity	Insulin response/Metabolic signaling	Promotes obesity-driven IR, compounding baseline PMOS metabolic severity	[72, 73]
*ERBB4*	Supports granulosa cell–oocyte paracrine communication, follicle development, and AMH production	ERBB4 signaling; Granulosa–oocyte junction; Hormonal regulatory pathways	Loss of function elevates AMH and androgens while suppressing folliculogenesis	[74, 75]

*AMH* anti-Müllerian hormone; *FSH* follicle-stimulating hormone; *GWAS* genome-wide association study; *IR* insulin resistance; *LH/CG* luteinizing hormone/choriogonadotropin; *PMOS* polyendocrine metabolic ovarian syndrome; *T2DM* type 2 diabetes mellitus

### Transcriptomics and Public Datasets Integration

Large public repositories, especially the Gene Expression Omnibus (GEO), have accelerated transcriptomic discoveries. Substantial alterations have been observed in granulosa cells in PMOS cases, including dysregulation of *TLR*, *NOD*, and *NOTCH* signalling pathways and downregulation of ovulation-related genes such as *HAS2* and *CBLN1* [76]. Integrated bioinformatics analysis identified thousands of differentially expressed genes involved in translation, respiratory electron transport, and transcription regulation, with hub genes *SAA1*, *ESR1*, and *NGFR* proposed as candidate biomarkers [77, 78]. Disrupted expression of *PGR*, *SIRT1*, and *ADAMTS1* in PMOS oocytes correlates with impaired ovulation and pregnancy outcomes [79]. Shared molecular signatures between PMOS and T2DM, including *BIRC3*, *DEPTOR*, *TNNL3*, and *ADRA2A,* further underscore overlapping metabolic pathways [80]. *CPEB4* has emerged as a mediator linking metabolic and ovarian dysfunction, with small-molecule modulators showing therapeutic potential in preclinical models [81].

### Non-coding RNA Networks

Non-coding RNAs (ncRNAs) serve as critical epigenetic rheostats in PMOS pathogenesis, fine-tuning the translational expression of major neuroendocrine and metabolic hubs. In adolescent phenotypes, elevated circulating microRNAs, specifically miR-15a-5p, miR-320-5p, miR-103a-5p, and miR-194-5p, closely track clinical metabolic dysregulation, offering high-fidelity diagnostic biomarkers before irreversible tissue remodeling occurs [82–84]. Beyond systemic circulation, deep sequencing reveals highly dysregulated long non-coding RNA (lncRNA) expressions within the serum, follicular fluid, and granulosa cell compartments [85]. When integrated into biological networks, LncRNA–miRNA–mRNA networks further illuminate mechanistic pathways. A XIST-centered competing endogenous RNA (ceRNA) axis (comprising *ETS2*, *AQP9, PLAU, PLEK, SOCS3,* and *TNFRSF1B*) directly bridges local granulosa cell inflammation with peripheral immune responses. Drug–gene interaction modeling of this specific axis points to folate/methotrexate and threonine pathways as actionable therapeutic targets [86]. In cumulus cells, the HOXA11-AS–miR-454-3p–CCND2 network tightly regulates cell cycle progression and estrogen receptor signalling. This specific axis functions as an absolute biomarker for ovarian dysfunction, effectively stratifying patients by their distinct follicular phenotypes [87]. By mapping these non-coding networks, multi-omics moves beyond static genetic mapping to capture the real-time, dynamic epigenetic state of the disease.

### Bridging Multi-Omics to Mechanism-Driven Interventions

The multi-omic data landscape of PMOS establishes that it is not a monolithic condition, but an intricate systems-level disorder requiring a dual-pronged therapeutic paradigm. As illustrated in our overall framework (Graphical abstract), multi-omics serves as the diagnostic and stratifying core that maps downstream cellular abnormalities and upstream genetic susceptibilities. These datasets do not merely describe pathology; they dictate the deployment of next-generation interventions. Upstream, multi-omic datasets reveal the specific genetic coordinates (*CYP17A1*, *DENND1A*, *INSR*) where high-fidelity CRISPR/Cas9 genome editors can act to functionally dissect or rewrite pathologically overactive networks. Downstream, multi-omics profiles the specific tissue niches—such as the pro-inflammatory, fibrotic ovarian ECM or dysregulated granulosa cell layers—that define the phenotypic targets for stem cell-derived exosomal therapies. Together, these multi-omic insights depict PMOS not as a single disease but as an intricate systems-level disorder that demands equally comprehensive, mechanism-based therapeutic strategies exploited by stem cell and CRISPR platforms.

## Stem Cell Research in PMOS

The multifactorial nature of PMOS encompasses chronic inflammation, extracellular matrix remodeling, disrupted folliculogenesis, metabolic dysfunction, and mitochondrial impairment. These features have driven interest in stem cell–based therapies for disease-modifying intervention [88, 89]. Among these, mesenchymal stem cells (MSCs) derived from bone marrow, adipose tissue, umbilical cord, and menstrual blood are particularly attractive due to their regenerative, anti-inflammatory, and immunomodulatory properties, with potential to restore ovarian function in PMOS [90, 91]. Their therapeutic effects are largely mediated through paracrine mechanisms. MSC-derived exosomes, which retain many bioactive molecules of the parent cells, reduce androgen levels, enhance Insulin sensitivity, and improve metabolic and reproductive outcomes in PMOS models [92, 93]. Thus, MSC-based interventions may bridge cellular dysfunction and functional ovarian recovery.

### Bone Marrow Mesenchymal Stem Cells: Harnessing Endogenous Immunomodulation

Bone marrow-derived MSCs (BM-MSCs) exhibit strong migration and proliferation in inflammatory environments, a condition highly characteristic of the PMOS ovary. Upon exposure to inflammatory stimuli such as LPS, they secrete potent cytokines and chemokines (VEGF-A, SDF-1α, IL-6, MCP-1), enabling immune modulation and recruitment of reparative cells [94, 95].

Beyond immunomodulation, BM-MSCs restore ovarian function by suppressing excessive autophagy in granulosa cells via activation of the PI3K/AKT/mTOR signaling pathway, thereby improving mitochondrial function, follicular quality, and endocrine balance [96]. Supplementation of in vitro maturation (IVM) media with BM-MSC–conditioned medium has been shown to improve oocyte cytoplasmic and nuclear maturation, fertilization rates, and embryo development in PMOS -like mice, marking a plausible innovative approach to address follicular arrest in PMOS [97].

In vivo studies further reinforce these findings. In letrozole-induced PMOS mice, BM-MSCs administration reduced ovarian inflammation, downregulated androgen-related genes via interleukin-10 (IL-10) secretion, and restored metabolic and reproductive functions, including fertility [98]. Bone morphogenetic protein 2 (BMP-2), a key component of the BM-MSC secretome, suppresses androgen synthesis, inhibits granulosa cell proliferation, and attenuates inflammation in vitro [99, 100].

Consistent with emerging evidence linking autophagy dysregulation and placental dysfunction in PMOS, MSC-based interventions normalize placental structure, enhance microvascular density, and improve pregnancy outcomes [96, 101]. Similarly, in testosterone-induced PMOS mice, BM-MSC transplantation improved folliculogenesis and ovarian morphology, increased FSH levels and antioxidant capacity, and reduced testosterone, LH, oxidative stress, and follicular apoptosis [102].

Building on these observations, MSC-derived exosomes demonstrate comparable therapeutic efficacy. In letrozole-induced PMOS mice, both intravenous and intraovarian administration of exosomes restored ovarian function and fertility while improving glucose metabolism. These findings supported FDA approval (IND 28896) for the first clinical trial of MSC-exosome therapy in reproductive disorders, advancing the development of cell-free treatment strategies [99].

### Adipose, Menstrual Blood-, and Placenta-Derived MSCs and Their Exosomes: Targeting Ovarian and Metabolic Pathways

Adipose-derived MSCs (AMSCs) offer an accessible and potent stem cell source. AMSCs and their exosomes deliver miR-323-3p, which enhances cumulus cell proliferation and inhibits apoptosis via Programmed cell death protein 4 (PDCD4); elevating miR-323-3p levels significantly improved ovarian function in a letrozole-induced PMOS mice [103]. Combination therapy with AMSCs with 2-α-naphthylmethyltrimethylammonium iodide (α-NETA), a Chemerin-like receptor 1 (CMKLR1) antagonist, improved metabolic and endocrine parameters, reduced circulating chemokines, downregulated adipose *CMKLR1* expression, and restored estrous cyclicity in PMOS rats [104]. AMSCs-derived exosomes enriched in miR-644-5p protect ovarian tissue by targeting p53, reducing granulosa cell apoptosis and inflammation in PMOS rats [105].

Human AMSC–based therapies may alleviate metabolic dysfunction by promoting brown adipose tissue (BAT) activation, reducing inflammation, and improving weight regulation, central features of PMOS pathophysiology [106, 107]. In cardiovascular studies, AMSC-derived exosomes promote macrophage polarization toward an anti-inflammatory phenotype and stimulate white-adipose browning via catecholamine-mediated pathways [108, 109], suggesting their relevance for mitigating chronic inflammation in PMOS.

Interestingly, AMSC-conditioned medium (AMSC-CM) may exert stronger therapeutic effects than the cells themselves. In PMOS rat models, AMSC-CM improved uterine structure, estrogen levels, and estrogen receptor expression [110]. In direct comparisons, they outperformed exosomes in restoring ovarian histology, hormone homeostasis, and fertility in letrozole-induced PMOS rats [111].

Menstrual blood–derived mesenchymal stem cells (Men-MSCs) also show therapeutic potential. In PMOS rat models, Men-MSCs and their exosomes enhance mitochondrial biogenesis, attenuate oxidative stress, and normalize estrogen production in granulosa cells [112]. In vivo administration of Men-MSCs improves ovarian histology by reducing follicular atresia and cyst formation while increasing the number of healthy follicles, accompanied by decreased fibrosis, regulated angiogenesis, and an improved ovarian microenvironment [113].

Placenta-derived mesenchymal stem cells (PD-MSCs) further broaden regenerative strategies by targeting both ovarian and systemic metabolic dysfunctions. In PMOS and ovariectomized rat models, PD-MSCs restore folliculogenesis, increase corpus luteum formation, reduce cystic follicles, and correct hormonal imbalances, including elevated LH and testosterone levels [114]. Additionally, PD-MSCs improve IR, lipid profiles, and liver function. These effects involve VEGF-driven angiogenesis and activation of the PI3K/AKT/mTOR and GSK3β/β-catenin signaling pathways, ultimately enhancing ovarian function [115].

Collectively, preclinical studies suggest that AMSC-, MenMSC-, and PD-MSC-based therapies may exert multifaceted effects on ovarian function, supporting further translational investigation in PMOS research.

### Umbilical Cord MSCs and Exosomes: Restoring Immune Homeostasis

Human umbilical cord MSCs (hUC-MSCs) and their exosomes exhibit strong anti-inflammatory properties. hUC-MSC–derived exosomes increase IL-10 levels while suppressing TNF-α and IFN-γ in granulosa cells from PMOS patients. Through inhibition of the NF-κB pathway, specifically by blocking phosphorylation and nuclear translocation of the p65 subunit, they reduce apoptosis and promote progesterone synthesis, improving ovarian function and fertility [116, 117].

Conditioned medium (CM) and EVs derived from human Wharton’s Jelly MSCs improve oocyte maturation, viability, morphology, and meiotic gene expression in both normal and PMOS-induced mice, rescuing impaired maturation and increased degeneration observed in PMOS oocytes [118, 119].

Complementing these findings, whole hUC-MSC transplantation similarly improves ovarian structure in DHEA-induced PMOS mice by reducing local and systemic inflammation. This treatment decreases pro-inflammatory cytokines (TNF-α, IL-1β, IFN-γ), lowers fibrosis-related gene expression, suppresses harmful immune cell populations (neutrophils, M1 macrophages, B cells, Th1 and Th17 cells), and increases regulatory T cells and M2 macrophages [120]. These findings underline the central role of immune rebalancing in MSC-mediated repair. However, while MSCs consistently improve ovarian structure in rodent models, variability in outcomes across hyperandrogenic versus metabolic phenotypes suggests phenotype-specific responsiveness, which remains insufficiently addressed in current studies.

### Induced Pluripotent Stem Cells: A Gateway to Personalized PMOS Modeling

Induced pluripotent stem cells (iPSCs), derived from reprogrammed somatic cells, represent a powerful platform for patient-specific disease modeling. PMOS patient-derived iPSCs differentiated into granulosa-like cells enable controlled investigations of dysregulated glucose metabolism, mitochondrial dysfunction, and altered metabolic, steroidogenic, and neurogenic pathways [121–124]. These models mirror clinical PMOS features, including elevated testosterone and estradiol levels [123, 124], offering unprecedented insight into cellular mechanisms and potential drug responses.

However, limitations include small sample sizes, poorly characterized cell populations, reliance on integrating viral vectors, insufficient controls, and limited functional validation. Ethical concerns and low differentiation efficiency further constrain translation [21].

### Current Challenges and Cell-Autonomous Barriers of Stem Cell Platforms

Despite substantial preclinical progress (Table 2), translating stem cell-based interventions into clinical therapies for PMOS remains constrained by interconnected biological, technical, and regulatory hurdles.

**Table 2:** Characterization of stem cell therapeutic platforms interrogated in preclinical models of PMOS

Stem Cell Type	Source/Model	Suggested Key Mechanism of Action	Therapeutic Outcome	Reference
BM-MSCs (Bone Marrow Mesenchymal Stem Cells)	Bone marrow/Rodent/in vitro human granulosa cells	Secretes paracrine IL-10, BMP-2, and anti-inflammatory cytokines; modulates PI3K/AKT/mTOR signaling to rescue mitochondrial function and reduce autophagy	Restores ovulation cycles, downregulates serum testosterone, improves follicular development, and normalizes placental microvascular density	[96, 100–102]
AD-MSCs (Adipose-Derived MSCs)	Adipose tissue/Rodent	Delivers miRNA-rich exosomes (e.g., miR-323-3p, miR-644-5p) targeting p53 and insulin pathways; downregulates adipose *CMKLR1*	Corrects metabolic profiles, improves insulin sensitivity, dampens inflammation, and restores estrous cyclicity	[105, 107, 109, 110]
Men-MSCs (Menstrual Blood-Derived Stem Cells)	Menstrual blood/Rodent	Promotes angiogenesis and attenuates oxidative stress; secretes VEGF and active MMPs	Rescues ovarian morphology, downregulates fibrosis, enhances vascularization, and increases follicle counts	[112, 113]
PD-MSCs (Placental-Derived Mesenchymal Stem Cell)	Placenta/Rodent	Modulates inflammation via VEGF paracrine delivery; activates GSK3β/β-catenin and PI3K pathways	Restores folliculogenesis, corrects hormone imbalances, and resolves lipid profiles and liver dysfunction	[114, 115]
UC-MSCs (Umbilical Cord Mesenchymal Stem Cells)	Umbilical cord/Rodent/In vitro human granulosa cells	Immunomodulation via NF-κB pathway blockade; downregulates pro-inflammatory TNF-α and IFN-γ	Minimizes endometrial fibrosis, improves endometrial receptivity, enhances implantation potential, and recovers fertility profiles	[116–120]
iPSCs (Induced Pluripotent Stem Cells)	Reprogrammed somatic cells/Human	Differentiates into stratified patient-specific granulosa-like or endometrial cells to map metabolic/steroidogenic defects	Establishes scalable, personalized in vitro disease modeling platforms	[21, 122–124]

*BMP-2* bone morphogenetic protein 2; *CMKLR1* chemerin-like receptor 1; *EV* extracellular vesicle; *IFN-γ* interferon gamma; *IL-10* interleukin 10; *iPSC*, induced pluripotent stem cell; *MMP* matrix metalloproteinase; *MSC* mesenchymal stem cell; *NF-κB* nuclear factor kappa B; *PMOS* polyendocrine metabolic ovarian syndrome; *TNF-α* tumor necrosis factor alpha; *VEGF* vascular endothelial growth factor

#### Discrepancies in Preclinical Models and the Human Translational Disconnect

Successful translation requires reconciling conflicting data from distinct chemical or endocrine animal models, each failing to fully replicate the complexity of human PMOS [125]. Letrozole models inhibit aromatase to block estrogen synthesis, creating an intraovarian microenvironment with robust follicular arrest, cystic follicles, and severe systemic metabolic phenotypes like insulin resistance and dyslipidemia. In contrast, DHEA models drive acute hyperandrogenism, resulting in localized ovarian changes such as granulosa cell apoptosis and follicular atresia, while lacking severe, chronic systemic metabolic dysfunction. Meanwhile, testosterone models disrupt neuroendocrine function through elevated LH pulsatility and peripheral metabolic traits, though the severity depends heavily on the exposure window and dosage. These acute, single-pathway rodent models cannot mirror the polygenic, multi-systemic, and heterogeneous human clinical subtypes that stem from lifelong genetic, epigenetic, and lifestyle factors [125, 126]. This mismatch explains why stem cell therapies showing high efficacy in animals frequently encounter profound barriers during human clinical validation [127].

#### Culture Drift and Secretome Instability in MSC Platforms

For adult stem cell platforms such as BM-MSCs, AMSCs, and hUC-MSCs, a primary challenge is the preservation of therapeutic potency during ex vivo expansion. Extended passaging frequently induces cellular senescence, causing a programmatic drift in their paracrine secretome profile [128]. When exposed to pathological host environments characterized by hyperandrogenism or severe chronic inflammation, the stability of MSC-derived therapeutic cargos (e.g., specific microRNAs like miR-323-3p or miR-644-5p) can become dysregulated. This functional volatility leads to unpredictable immunomodulatory responses and inconsistent recovery of downstream granulosa or ovarian architectural components [129].

## CRISPR/Cas9 Technology and Its Application in PMOS Pathology

While stem cell therapies address downstream tissue dysfunction, CRISPR enables upstream mechanistic interrogation of these pathways. CRISPR (Clustered Regularly Interspaced Short Palindromic Repeats)/Cas9, originally discovered as a bacterial immune defense system, has become a transformative genome-editing platform [130]. Guided by RNA (gRNA), Cas9 introduces site-specific DNA breaks that enable gene disruption, correction, or insertion with high precision via endogenous repair pathways [131]. Its precision and efficiency have made CRISPR central to functional genomics in endocrine and metabolic research [132, 133].

PMOS, positioned at the intersection of reproductive and metabolic dysfunction [22], represents a compelling context for CRISPR-based functional interrogation. While potential therapeutic applications remain conceptual, current strategies focus on somatic, non-heritable editing approaches, including ex vivo gene correction and tissue-targeted in vivo modulation. Given the polygenic architecture of PMOS, characterized by numerous small-effect variants interacting with environmental and epigenetic factors, CRISPR is best positioned as a tool for functional validation, pathway interrogation, and disease modeling, rather than as a direct curative intervention (Fig. 1C, D). Table 3, hypothetically, summarizes representative genes and pathways interrogated using genetic editing approaches and their hypothesized relevance to PMOS pathogenesis.

**Table 3:** Summary of candidate risk genes and molecular pathways interrogated via genetic editing as basic research tools to map PMOS pathogenesis

Biological Hallmark	Gene(s)	Primary Pathophysiological Role	Experimental CRISPR Tool	Predicted Research Impact	Reference
Hyperandrogenism	*CYP17A1*	Drives ovarian theca-cell steroidogenesis	Knockout or CRISPRi transcriptional repression	Suppresses local androgen biosynthesis	[51, 69]
	*DENND1A*	Facilitates endosomal trafficking; DENND1A.V2 isoform drives androgen excess	CRISPRi against variant V2/CRISPRa for homeostatic balancing	Reduces testosterone overproduction in theca cells	[52, 57]
	*THADA*	Coordinates cell death and energy homeostasis	CRISPR gene correction or transcriptomic rescue	Assesses limits of single vs. polygenic models	[57, 62]
Insulin Resistance & Metabolic Dysfunction	*INSR, IRS1*	Regulate classical insulin receptor tyrosine kinase signaling cascades	CRISPR gene editing to restore signaling fidelity	Reverses insulin insensitivity and restores glucose profile	[49, 50, 65]
	*FKBP5*	Regulates stress hormones linked to insulin resistance	CRISPRi or target knockdown to suppress FKBP5 signaling	Ameliorates adipose-tissue inflammation	[37]
	*RAB5B*	Dictates endocytic trafficking and insulin receptor recycling	CRISPR gene correction or regulatory expression tuning	Restores insulin-receptor membrane recycling kinetics	[37, 38]
	*LEPR*	Mediates leptin signaling across metabolic and reproductive tissues	CRISPRa to upregulate receptor density profiles	Restores metabolic homeostasis and gonadotropin regulation	[37]
Follicular Arrest & Hyper-Responsiveness	*FSHR*	Governs follicle maturation	CRISPRa targeted transcriptional overactivation screening	Restores folliculogenesis and granulosa cell function	[134, 135]
	*YAP1*	Core Hippo signaling pathway effector driving follicle selection	CRISPRi to repress overactive YAP1 expression	Attenuates follicular arrest by balancing cell expansion	[67, 68]
	*LHCGR*	Mediates LH/CG signaling in ovarian cells	CRISPRi or regulatory knockdown (complete KO impairs ovulation)	Normalizes LH signaling loops to resolve follicular arrest	[58, 59]
	*ERBB4*	Coordinates paracrine crosstalk at granulosa–oocyte junctions	Somatic gene knockout or regulatory alteration	Evaluates AMH hypersecretion and cycle irregularity	[74, 75]
Oxidative Stress & Cellular Repair	*SOD2*	Functions as a key mitochondrial antioxidant enzyme	CRISPRa to enhance endogenous antioxidant capacity	Minimizes oxidative damage, improves granulosa cell survival	[37, 38]
	*WWTR1 (TAZ)*	Functions as a Hippo path co-activator; controls tissue growth	CRISPRi to diminish structural pathoveractivation	Restores functional balances across granulosa-theca cell borders	[35, 36]
	*CHEK2*	Governs DNA damage checkpoints and intrinsic repair cascades	Somatic gene knockout or CRISPRa validation profiling	Protects functional follicle pools from depletion	[35]
Adipokine Signaling & Endocrine Crosstalk	*ADIPOQ*	Encodes adiponectin to enhance tissue insulin action	CRISPR-mediated regulatory modulation of promoters	Stabilizes glucose capture while suppressing inflammation	[48, 121]
	*ADIPOR1*	Binds adiponectin to stimulate the AMPK pathway	CRISPRa to elevate receptor expression	Optimizes metabolic signaling alongside ovarian function	[121, 122]
Endometrial Receptivity Barriers	*PGR*	Coordinates progesterone receptor transcription	CRISPRa to elevate receptor activity	Restores decidualization markers and uterine receptivity	[136, 137]
	*FOXO1*	Modulates uterine receptivity	CRISPRa to augment baseline transcriptional activity	Re-establishes endometrial signaling networks	[30, 137]
	*HOXA10*	Modulates uterine receptivity	CRISPRa targeted upregulation during the window of receptivity	Enhances blastocyst implantation	[30, 137]
Mitochondrial Competence Loss	*TFAM*	Supports mitochondrial DNA structural transcription and mass	CRISPRa to reinforce mitochondrial function	Elevates ATP production and improves oocyte quality	[37, 121, 122]
	*POLG*	Drives mitochondrial DNA replication	High-fidelity somatic correction or CRISPRa activation	Increases mitochondrial DNA copy numbers	[37, 122]
	*NRF1*	Triggers nuclear respiratory pathways for organelle biogenesis	CRISPRa to drive downstream transcription networks	Restores oocyte metabolic competence and structural integrity	[37, 121]
Granulosa Cell Loss Loops	*TOX3*	Regulates chromatin structure and cell-cycle progression	CRISPRi to selectively downregulate apoptotic triggers	Prevents granulosa cell apoptosis, supporting follicle growth	[77, 78]

*AMH* anti-Müllerian hormone; *AMPK* AMP-activated protein kinase; *ATP* adenosine triphosphate; *CG* chorionic gonadotropin; *CRISPRa* CRISPR activation; *CRISPRi* CRISPR interference; *IR* insulin resistance; *KO* knockout; *LH* luteinizing hormone; *mtDNA* mitochondrial DNA

### From Genetic Clues to Functional Proof: CRISPR Validation of PMOS Risk Loci

Although GWAS studies have identified numerous susceptibility loci, association alone does not establish causation [138]. CRISPR-based approaches enable direct functional interrogation of candidate genes and regulatory elements. Targeted perturbation studies have clarified the roles of genes such as *DENND1A*, *THADA*, *FSHB*, and *GATA4* in disease-related pathways (Table 1). For instance, *SH2B3* (LNK) knockout mice showed improved estrous cyclicity and glucose metabolism in PMOS-like conditions [139]. In contrast, the failure of *THADA* deletion to induce PMOS-like phenotypes in rodent models, despite its robust genome-wide association, underscores the key translational disconnect between statistical genetic mapping and physiological execution [140]. This incongruence may stem from human-specific evolutionary metabolic adaptations or cell-autonomous regulatory loops that are poorly recapitulated in baseline murine physiology. Similarly, depletion of *IGFBP7* attenuated DHEA-induced PMOS features, highlighting an immune-related mechanism [141]. Similarly, regulatory editing of *DENND1A* demonstrates how noncoding variants can influence androgen production [142]. Complementary genome-wide screens in sheep granulosa cells identified FSH-responsive transcriptional networks [134]. These findings reinforce the importance of CRISPR in distinguishing causal mechanisms from statistical associations.

### Epigenetic Rewriting: CRISPR/dCas9 and TET-based Epigenome Editors

CRISPR-based epigenome editors, created by fusing catalytically inactive Cas9 (dCas9) to transcriptional or epigenetic modifiers, allow gene activation or repression without altering DNA sequence [143]. Targeting aberrant methylation patterns in genes such as *AMHR2, INSR, PPARGC1A*, *LHCGR*, and *CYP19A1* provides a precise method to investigate regulatory dysregulation in PMOS [144, 145]. For example, demethylation of the *CYP19A1* promoter could restore aromatase expression, potentially improving estrogen synthesis [146], while activation of *INSR* may enhance insulin sensitivity [147]. Editing nuclear-encoded mitochondrial regulators further clarifies links between epigenetic remodeling and metabolic dysfunction [146]. These reversible approaches offer refined tools for dissecting gene regulation, although their therapeutic application remains exploratory.

### Editing Neuroendocrine Networks: CRISPR Approaches to Normalize GnRH Pulsatility

CRISPR-based genome editing enables precise investigation of hypothalamic circuits that control GnRH and LH pulsatility, a central feature of PMOS pathophysiology. Dysregulation of KNDy neurons (Kisspeptin/Neurokinin B/Dynorphin) drives the abnormally rapid GnRH pulse frequency observed in PMOS, making these neurons ideal targets for mechanistic studies[148, 149]. Using AAV-delivered CRISPR systems, researchers can achieve region-specific gene editing in post-mitotic neurons, with recent studies demonstrating successful targeting of neuronal receptor genes, such as the oxytocin receptor, across multiple rodent species [150, 151]. Functional applications include CRISPR/Cas9 or CRISPRi-mediated disruption of *Kiss1*, *Tac3/Tacr3*, androgen receptor (*AR*) to test how altered neuronal androgen sensitivity or metabolic signaling contributes to PMOS-like hyperandrogenism, anovulation, and LH excess [152, 153]. These studies allow high-resolution mapping of neuroendocrine circuitry underlying hyperandrogenism and anovulation, identifying nodal regulatory points without implying immediate clinical correction.

### Restoring Ovarian Function: CRISPR-mediated Genome Editing in Ovarian Cells

CRISPR has been used to investigate ovarian signaling pathways, including gonadotropin receptor function and steroidogenesis in vivo and in vitro. In adult female mice, CRISPR-mediated gonadotrope depletion reveals that FSH and LH regulate not only gonadal function but also metabolic and hepatic health, uncovering a pituitary hormone–to–hormone communication pathway in which FSH acts on corticotropes to restrain corticosterone production and prevent fatty liver [154]. Similarly, CRISPR-mediated *FSHR* expression in human granulosa cells increased FSH sensitivity and amplified downstream signaling [135]. In vivo, rodent models engineered via CRISPR, including luteinizing hormone/choriogonadotropin receptor (*Lhcgr) knockout* mice, have provided insights into LH-driven hyperandrogenism and follicular arrest [155, 156], while LHCGR signaling modulation holds potential for restoring ovulation without systemic hormonal side effects. Similarly, targeted editing of *ERBB4* in granulosa cells allows investigation of ovulatory dysfunction, while postnatal CRISPRi of *Cyp17a1* in CYP17-overexpressing mice suppresses androgen production [151]. Knock-in strategies, such as introducing human *DENND1A* SNPs into regulatory regions, enable precise modeling of gene regulation and steroidogenesis [157]. In Goto-Kakizaki (GK) rats, CRISPR knockouts of *amh, lhcgr, and insr* allowed dissection of reproductive versus metabolic phenotypes [126, 158]. These approaches may improve the mechanistic resolution of gene–hormone interactions.

### Modeling PMOS Pathophysiology Using iPSCs: CRISPR-based Organoid and In Vivo Approaches

Integration of CRISPR with patient-derived iPSCs has enabled the development of genetically defined disease models. Isogenic iPSC systems allow controlled investigation of gene–phenotype relationships in relevant cell types, including granulosa and endometrial cells. Adult stem cell- and iPSC-derived organoids further enhance physiological relevance by recapitulating the 3D architecture, epithelial–mesenchymal interactions, and dynamic hormone responsiveness of human reproductive tissues. CRISPR enables rapid generation of isogenic organoid pairs to interrogate the functional consequences of disease-driving alleles and evaluate gene correction strategies [159]. Scaffold-free epithelial endometrial organoids derived from PMOS donors have proven particularly valuable for modeling aberrant responses to estradiol and progesterone, dysregulated inflammatory signaling, altered insulin and androgen sensitivity, and implantation-related defects. These platforms also serve as ex vivo testbeds for evaluating CRISPR editing efficiency, off-target safety, and long-term genomic stability before clinical application [160, 161].

Beyond in vitro models, CRISPR-edited iPSCs and organoids can be incorporated into in vivo models, including xenograft transplantation into immunodeficient mice, to interrogate tissue-level physiology within integrated endocrine metabolic interactions. Such approaches enable assessments of how PMOS-associated genomic perturbations influence endocrine feedback loops, folliculogenesis, implantation competence, and systemic metabolic homeostasis, which cannot be fully captured in isolated cultures [162, 163]. Overall, while gene-corrected organoids hold therapeutic potential, particularly in addressing infertility and metabolic health challenges associated with PMOS, their primary strength lies in mechanistic discovery and translational modelling [164, 165].

### Targeting Ovarian Fibrosis and Mechanical Signaling: CRISPR Editing of Extracellular Matrix

Ovarian fibrosis and altered ECM composition are increasingly recognized as contributors to follicular arrest and ovulatory dysfunction in PMOS. Genetic manipulation of ECM–related genes, including collagen I/III, fibronectin, and regulators of matrix turnover, has long been used to interrogate tissue remodeling pathways, with CRISPR/Cas9 now providing a more efficient means of implementing such perturbations in defined cellular contexts [166]. Precise genome editing in pluripotent or mesenchymal stem cell models enables functional interrogation and correction of ECM dysregulation, offering a promising strategy to normalize ovarian microenvironmental cues [167].

Targeted modification of genes controlling collagen deposition, fibronectin expression, or matrix metalloproteinase (MMP) activity has shown that excessive ECM accumulation limits follicular expansion and compromises ovulatory competence, providing a mechanistic link between fibrosis and reproductive dysfunction in PMOS [34, 167]. Although direct CRISPR applications of ECM regulation in PMOS remain limited, evidence from related ovarian pathologies supports the feasibility of this approach. For example, CRISPR-mediated knockdown of *TIMP-2*, an inhibitor of MMP activity, in ovarian cancer cell models altered ECM dynamics and validated the functional importance of matrix-remodeling pathways independent of disease context [168]. Looking ahead, cell-specific CRISPR delivery to granulosa or theca cells via nanocarriers or exosome-based systems could enable targeted correction of ECM abnormalities, potentially reversing ovarian fibrosis, restoring follicular competence, and improving reproductive and metabolic outcomes in PMOS.

### Correcting Endocrine-Metabolic Crosstalk: CRISPR for Alleviating Insulin Resistance

Beyond reproduction, CRISPR-based genome editing is rapidly transforming metabolic research [169, 170] and offers new opportunities to dissect IR, adipocyte dysfunction, and energy homeostasis pathways relevant to PMOS. Importantly, many of these insights build on earlier knockout models, with CRISPR primarily improving efficiency and species accessibility rather than redefining conceptual frameworks. Global elimination of *IRS2* in mice results in combined peripheral IR and impaired pancreatic β-cell compensation, leading to hyperglycemia and progressive T2DM, thereby establishing a causal role for IRS2 in maintaining insulin-β-cell homeostasis [171, 172].

More recently, a CRISPR/Cas9-mediated knockout of *IRS2* in the golden (Syrian) hamster established a non-obese T2DM model characterized by β-cell hypoplasia, hyperglycemia, and impaired glucose tolerance, without dietary or pharmacological interventions [171, 173, 174]. While not a PMOS model per se, these studies clarify how intrinsic defects in Insulin signaling can drive metabolic dysfunction independently of obesity, a feature highly relevant to PMOS. In parallel, CRISPR/Cas9 has recently been applied to modulate ceRNA networks, including lncRNAs, miRNAs, and mRNAs implicated in IR [175]. Collectively, these approaches highlight how precise genetic manipulation can be used to interrogate metabolic control nodes implicated in PMOS-associated IR, rather than positioning CRISPR itself as a disease-corrective modality [175, 176].

### Modulation of Adipocyte Differentiation: CRISPR for Adipocyte Dysfunction

CRISPR studies targeting genes (*IRS1*, *PDGFC, FST, FKBP5*, and *PPARG)* in preadipocytes reveal new mechanisms governing adipogenesis, lipid handling, and insulin signaling [177]. In a proof-of-concept study, CRISPR/Cas9-mediated knock out of *FKBP5* and *PPARG* in primary human preadipocytes demonstrated distinct functional outcomes. With high efficiency editing (> 90%), loss of *PPARG* abolished adipocyte differentiation, confirming its established role as a master regulator of adipogenesis and validating the experimental system rather than revealing a novel mechanism. In contrast, deletion of *FKBP5* altered glucocorticoid-mediated regulation of glucose metabolism, implicating this gene in stress-hormone insulin signaling cross-talk [178], a pathway increasingly recognized as relevant to metabolic dysfunction in PMOS [9].

Beyond loss-of-function approaches, CRISPR-based gene activation (CRISPRa) has been used to upregulate energy metabolism–related genes. Activation of muscle-derived myokines *FGF21* and *FNDC5* promoted adipocyte browning and improved glucose tolerance in diet-induced obese mice, illustrating how modulation of inter-tissue signaling pathways can influence systemic metabolic homeostasis [179]. In parallel, suppression of *NRIP1* in adipocytes enhanced expression of the thermogenic marker *UCP1* and promoted browning/beiging of white adipocytes, an effect that increases energy expenditure and may improve metabolic homeostasis [180]. While these studies were not conducted in PMOS models, they provide conceptual frameworks for understanding how adipocyte plasticity may be leveraged to counteract metabolic dysfunction associated with PMOS.

### Resolving Chronic Inflammation: CRISPR-mediated Immune and Cytokine Pathway Correction

CRISPR interference (CRISPRi), using catalytically inactive Cas9 fused to transcriptional repressor domains, has been applied to silence pro-inflammatory cytokine genes such as *IL-6, IFN-γ*, and *CD40* in human immune cells, resulting in sustained suppression of cytokine release and immune activation [181]. These studies demonstrate the feasibility of selectively dampening inflammatory signaling, rather than establishing disease correction. In parallel, CRISPR-mediated epigenetic editing has enabled reprogramming of immune responses by modifying DNA methylation at regulatory loci. A recent 2025 study demonstrated that targeted methylation or demethylation of the *IL1RN* promoter using CRISPR–dCas9–TET1 or –DNMT3A significantly altered inflammatory signaling and cell fate in human myeloid cells [182].

CRISPR-engineered stem and immune cells are also being investigated to enhance anti-inflammatory activity or improve cell survival in inflammatory environments. For example, repression of inflammatory receptors such as *TNFR1* and *IL1R1* has been shown to improve stem cell survival without compromising immunomodulatory function [183]. However, challenges remain, as CRISPR/Cas9 editing in hematopoietic stem and progenitor cells can induce DNA damage responses and inflammatory signaling, potentially leading to senescence or functional impairment [184].

Although many of these approaches remain largely preclinical, they provide experimental tools to dissect immune-metabolic and cytokine-driven mechanisms that are highly relevant to PMOS. Applied cautiously, such approaches may help clarify how chronic inflammation contributes to IR, androgen excess, and ovarian dysfunction, thereby informing future mechanistic studies.

### Reinstating Endometrial Receptivity: CRISPR for Progesterone Resistance and Implantation

Progesterone resistance, characterized by altered expression of progesterone receptors (PR) (e.g., aberrant PRA/PRB ratio in uterine epithelium *vs.* stroma) and impaired regulation of classical PR-target genes (e.g., *LIF*, *CLDN-4*), is increasingly recognized as a major barrier to endometrial receptivity and implantation in women with PMOS [28, 185, 186]. In addition to defective PR signaling, dysregulation of metabolic pathways, mitochondrial function, inflammatory tone, and adhesion molecule expression further compromises endometrial function, underscoring the multifactorial nature of implantation failure in PMOS [187, 188].

Genetic manipulation approaches, now frequently implemented using CRISPR/Cas9, enable targeted interrogation of PR signaling components and downstream effectors in controlled experimental systems. For example, CRISPR/Cas9 knockout of *ARID1A* in endometrial cancer cells can induce progesterone resistance through downregulation of the PR expression, establishing a causal link between chromatin remodeling and hormone responsiveness [189]. Building on such mechanistic insight, donor-derived iPSC models have been used to generate endometrial stromal cells in which progesterone signaling pathways can be genetically modulated or corrected, allowing direct assessment of how restoring PR responsiveness affects decidualization and implantation-related gene expression [136, 137]. Moreover, implantation marker genes (*HOXA10*, *HOXA11*, *LIF*, *STC1*) can be edited to test their functional contribution to endometrial receptivity [190–192]. For instance, modulation of *STC1* expression has been shown to influence implantation-related processes, suggesting that altered expression of this gene contributes to defective endometrial signaling in PMOS rather than serving as a standalone therapeutic target [29, 190, 191], highlighting another avenue by which gene editing could address infertility in PMOS.

### Mitochondrial Genomics Corrections: A CRISPR-Powered Perspective into Mitochondrial Defects

Mitochondrial abnormalities are increasingly implicated in hyperandrogenism, IR, impaired oocyte competence, and endometrial function [37]. Leveraging CRISPR to target nuclear-encoded mitochondrial genes (*TFAM*, *POLG*, and *NRF1*) offers a promising strategy for modeling and potentially treating mitochondrial dysfunction in PMOS pathophysiology [193]. While mitochondrial DNA itself remains difficult to edit, nuclear-encoded mitochondrial genes are readily accessible to conventional genome-editing approaches, including CRISPR/Cas9, allowing controlled manipulation in both in vitro and in vivo models [194].

Targeted disruption or modulation of these genes can provide insight into how altered mitochondrial biogenesis, oxidative phosphorylation, and reactive oxygen species handling influence androgen production, follicular development, and Insulin signaling [195]. Importantly, these studies primarily serve to define causal links between mitochondrial dysfunction and PMOS phenotypes, rather than to establish immediate therapeutic strategies. Although CRISPR-mediated interventions have theoretical potential to enhance mitochondrial integrity and metabolic homeostasis, such applications remain exploratory. At present, mitochondrial-focused genome editing is best viewed as a mechanistic tool to understand how cellular energy metabolism intersects with reproductive and metabolic dysfunction in PMOS [196].

### CRISPR Meets the Microbiome: Mapping the PMOS Gut–Ovary Axis

Growing evidence links gut dysbiosis with endocrine, metabolic, and inflammatory abnormalities in PMOS, highlighting the gut–ovary axis as a contributor to disease heterogeneity [197, 198]. In fact, alterations in the gut microbiome correlate directly with PMOS phenotype [199]. Functional Genomics approaches, including CRISPR-based perturbation of host genes, offer a means to interrogate how host genetic variation shapes microbial composition, immune signaling, and metabolic pathways that influence ovarian function in PMOS [200].

At the same time, stem cell–based systems provide complementary insight by revealing how paracrine signaling, immunomodulation, and metabolic regulation may indirectly influence microbiome stability and host–microbe interactions [201, 202]. Conceptually, integrating CRISPR-guided pathway mapping with stem-cell–derived biological signals provides a systems-level approach to addressing the multifactorial nature of PMOS, linking genomic dysregulation, microbial imbalance, and endocrine dysfunction within a unified therapeutic landscape.

### CRISPR-Nanoparticle Delivery: Technical Considerations Toward Precision Therapies

The heterogeneous and chronic symptoms of PMOS often limit the efficacy of current treatments, including hormonal therapies and insulin-sensitizing agents [203]. In contrast, nanomedicine offers a promising alternative by enhancing precision, efficacy, and minimizing side effects, particularly in drug delivery, gene modulation, and biomarker detection [204].

Integrating CRISPR technology into nanoparticle systems represents a major step towards next-generation PMOS therapies. Non-viral nanocarriers, for example, lipid nanoparticles (LNPs), polymeric nanoparticles, or other synthetic nanocarriers, have been demonstrated to deliver CRISPR/Cas9 components (Cas9 mRNA or protein and guide RNAs) both in vitro and in vivo. These systems afford efficient genome editing while minimizing immunogenicity and off-target effects [205, 206]. Moreover, polymer-based and hybrid nanoparticle formulations have been engineered to co-deliver CRISPR components alongside therapeutic cargos, enabling combinatorial therapies that can both correct gene-level dysregulation and modulate pathological pathways [207].

Although such CRISPR–nanoparticle approaches have not yet been applied directly to PMOS models, related studies using nanoparticle-delivered antioxidants or anti-inflammatory agents in ovarian and reproductive disorders demonstrate the feasibility of targeted delivery [208]. Synergistic approaches, such as combining CRISPR silencing of inflammatory pathways with nanoparticle delivery of anti-inflammatory or antioxidant agents, may address hormonal, inflammatory, and metabolic dysfunction simultaneously, as observed in treating T2DM [209]. At present, these approaches remain preclinical and should be framed as platform technologies that may support future PMOS research rather than as near-term therapies [205].

## Multiplex CRISPR Engineering and iPSC Models for Polygenic Trait Dissection: A More Representative Platform than Gene Editing

Recent advances in multiplex and high-throughput CRISPR technologies now permit the simultaneous perturbation of dozens to hundreds of genomic loci, offering a more realistic experimental framework for modeling polygenic traits than traditional single-gene editing [210, 211]. Pooled gRNA libraries, saturation genome editing (SGE), multiplex base editing, and scalable prime editing platforms enable parallel functional interrogation of large variant sets, generation of endogenous variant effect maps, and combinatorial perturbation screens in mammalian cells [212, 213]. These approaches represent major methodological progress towards dissecting the distributed genetic architecture underlying complex disorders [214, 215]. However, for highly polygenic conditions such as PMOS, where ~ 100 GWAS loci explain only a modest fraction of heritability and most common variants exert small individual effects, editing a limited subset of genes is unlikely to recapitulate the full disease phenotype [44]. While rare monogenic or oligogenic PMOS forms may constitute tractable targets for precise genome editing, the broader syndrome reflects cumulative, small-effect variation across regulatory and metabolic networks. In this context, iPSC lines derived from individuals with high polygenic risk scores provide a more representative and physiologically integrated platform, capturing the combined influence of inherited variants without the artifacts introduced by artificial single-gene manipulation. As discussed in our previous review [21], leveraging genetically enriched iPSC models alongside multiplex CRISPR screening offers a complementary strategy for mechanistic discovery, with greater relevance for complex trait biology than therapeutic gene correction alone [216].

## Translational Limitations and Future Challenges in CRISPR and Stem Cell Applications

### Biological Complexity and Disease Heterogeneity

Although CRISPR-Cas9 holds promise for targeting PMOS-associated genes such as *DENND1A*, *LHCGR*, and *FSHR*, its application in elucidating disease mechanisms and developing therapeutic strategies, particularly for PMOS-related infertility, remains largely confined to preclinical models. Clinical translation is hindered by the polygenic and multifactorial nature of PMOS, driven by intertwined genetic, metabolic, hormonal, and environmental influences [217]. This limits the effectiveness of single-gene editing approaches and complicates precision medicine approaches [218]. In addition, pronounced phenotypic heterogeneity, including hyperandrogenic and metabolic subtypes, further limits the development of universal therapeutic strategies [219, 220]. These diverse molecular drivers necessitate personalized and context-specific interventions rather than one-size-fits-all approaches.

### Technical and Delivery Challenges

Technical constraints represent a major obstacle for both CRISPR and stem cell platforms. Off-target edits and unintended genomic alterations remain significant risks, especially in a reproductive context where germline modifications could have transgenerational consequences [221].

Efficient and tissue-specific delivery remains equally challenging. While nanoparticle-based delivery systems may improve tissue specificity, such as targeting ovarian theca cells or adipocytes, achieving efficient, precise delivery to ovarian or hypothalamic tissues remains a major obstacle [205, 222]. Achieving controlled, safe, and reproducible in vivo editing is therefore a critical unmet need. Emerging strategies such as transient gene modulation (e.g., CRISPR-based repression of *CYP17A1* or *AMHR2*) may allow reversible, cycle-dependent interventions, but their clinical feasibility requires further validation.

### Safety, Stability, and Knowledge Gaps

Significant safety concerns continue to restrict clinical translation. Genome-editing approaches may induce DNA damage responses, genomic instability, or immune activation, while stem cell–based therapies carry risks of tumorigenesis and long-term functional variability [219]. Moreover, knowledge gaps in PMOS biology remain substantial. Most studies are based on short-term experiments in homogeneous populations, limiting understanding of long-term safety, durability, and population-level variability [20, 39, 219]. These limitations highlight the need for longitudinal studies and diverse clinical cohorts [220, 221].

### Epigenetic Editing: Opportunities and Constraints

Epigenetic CRISPR tools offer additional opportunities but also face challenges. The efficiency of CRISPR-dCas9–mediated methylation or histone modification is highly dependent on local chromatin structure; dense heterochromatin can hinder targeting, while the dynamic nature of epigenetic regulation complicates prediction of editing outcomes [223]. For example, densely packed heterochromatin can create steric hindrance, limiting access to target DNA sequences and reducing editing efficiency. Moreover, the landscape of epigenetic regulation is inherently complex and dynamic, shaped by intricate gene–gene interactions and environmental factors [224]. While CRISPR-dCas9 systems can induce specific changes such as DNA methylation or histone modifications, the reversibility and long-term stability of these modifications remain significant challenges. Some alterations may persist despite environmental changes, while natural cellular mechanisms may undo others or require additional targeted interventions [225].

### Ethical, Regulatory, and Translational Landscape

The application of genome editing and regenerative therapies in reproductive medicine raises profound ethical and regulatory challenges. Germline editing remains prohibited due to its heritable consequences and societal implications [226, 227], while somatic interventions require rigorous informed consent and risk assessment [228]. Furthermore, the high cost and technical complexity of these approaches risk exacerbating existing healthcare inequities, underscoring the need for equitable access and responsible governance.

Translating CRISPR/Cas9- and stem cell-based approaches from experimental systems to clinical settings remains challenging, particularly for polygenic and heterogeneous disorders such as PMOS [229]. While early clinical trials in monogenic diseases demonstrate feasibility and safety [230], PMOS currently lacks clearly defined, single-gene targets suitable for direct therapeutic editing. Consequently, CRISPR applications in PMOS are currently best positioned as tools for functional genomics, disease modeling, and pathway validation, rather than immediate therapeutic interventions.

Progress in this field will require stringent regulatory oversight, standardized experimental frameworks, and transparent reporting of both benefits and risks. Public engagement and international consensus on ethical standards will be essential to foster trust and guide responsible innovation. Within this context (Table 4), CRISPR technologies hold significant value in advancing mechanistic understanding and informing the development of safer, more targeted strategies [165, 231].

**Table 4:** Key translational, biological, and technical bottlenecks challenging the application of CRISPR platforms and stem cell therapies in PMOS

Translational Challenge	Primary Biological/Technical Bottleneck	Proposed Mitigation & Optimization Strategy
Off-Target CRISPR Effects	Unintended genomic alterations; risk of malignant transformation	• Deploy ultra-high-fidelity variants (e.g., HiFi-Cas9) • Implement paired dCas9 nicking • Validate via genome-wide screening (e.g., GUIDE-seq)
Stem Cell Tumorigenicity	Uncontrolled proliferation, genomic drift, or teratoma formation	• Enforce strict pre-transplantation verification • Screen for post-expansion genomic stability • Shift toward cell-free, exosome-based platforms
PMOS Heterogeneity	Diverse clinical phenotypes (hyperandrogenic, metabolic, and inflammatory) hinder universal protocols	• Adopt multi-omics patient stratification • Leverage AI pipelines for patient-treatment matching
In Vivo Delivery Barriers	Poor tissue-homing and inadequate targeting of ovaries, endometrium, or adipose tissues	• Develop tissue-homing biomimetic nanocarriers • Bioengineer target-specific exosome vectors • Optimize localized intraovarian administration
Ethical & Regulatory Friction	Concerns over embryo use, germline editing, and unregulated clinics	• Establish global regulatory/safety guidelines • Limit editing strictly to non-heritable somatic targets • Conduct rigorous, double-blind randomized trials

*AI* artificial intelligence; *CRISPR* clustered regularly interspaced short palindromic repeats; *dCas9* catalytically inactive Cas9; *PMOS* polyendocrine metabolic ovarian syndrome

## Conclusion, Future Directions, and Recommendations

PMOS is a complex disorder driven by intertwined genetic, metabolic, and endocrine mechanisms. Current treatments remain largely symptomatic, highlighting the need for mechanism-based approaches. Stem cell–based platforms and CRISPR/Cas9 technologies have emerged as complementary tools with distinct but interconnected roles. Stem cell–based approaches demonstrate promising regenerative and immunomodulatory potential, with the capacity to restore ovarian structure, improve metabolic homeostasis, and modulate inflammatory pathways in preclinical models. In parallel, CRISPR technologies provide an unparalleled framework for functional genomics, enabling the precise interrogation of disease-associated genes, regulatory elements, and signaling networks that underlie PMOS pathophysiology.

However, the translational potential of these approaches remains limited. The highly polygenic and heterogeneous nature of PMOS complicates the development of universal therapeutic strategies, while technical challenges—including targeted delivery, off-target effects, and long-term safety—continue to restrict clinical application. Ethical and regulatory considerations, particularly in the context of reproductive medicine, further underscore the need for cautious and responsible advancement.

Importantly, the greatest near-term impact of these technologies is likely to lie in disease modeling, mechanistic discovery, and biomarker identification, rather than direct therapeutic intervention. The integration of CRISPR-edited iPSC models, organoid systems, and multi-omics datasets offers a powerful platform for dissecting complex disease mechanisms, enabling the identification of clinically actionable targets.

Future research should adopt system-level, integrative approaches that combine genomics, transcriptomics, metabolomics, and microbiome data with advanced computational tools, including artificial intelligence and machine learning [232]. The development of three-dimensional ovarian and endometrial organoids for preclinical testing [233], alongside AI-driven CRISPR optimization [234], will be essential to harmonize ethical governance, regulatory oversight, and equitable access to emerging biotechnologies. These strategies will facilitate patient stratification, improve predictive modeling, and support the development of precision medicine frameworks tailored to distinct PMOS phenotypes.

In summary, while CRISPR- and stem cell–based technologies are unlikely to provide an immediate cure for PMOS, they represent an indispensable component for the next-generation research ecosystem. Their continued development, guided by rigorous scientific validation, ethical oversight, and interdisciplinary collaboration, will be critical for transforming our understanding of PMOS and advancing toward mechanism-based, personalized therapeutic strategies.

## Data Availability

No datasets were generated or analysed during the current study.

## Declarations

### Search Methods

The review was written after a thorough search of the literature using the terms “polycystic ovarian syndrome/polyendocrine metabolic ovarian syndrome”, “genome-wide association study”, “CRISPR/Cas9”, “human embryonic stem cells”, “human induced pluripotent stem cells”, “mesenchymal stem cells” in the PubMed database. Only articles written in English were included.
